# Experience-dependent functional plasticity and visual response selectivity of surviving subplate neurons in the mouse visual cortex

**DOI:** 10.1073/pnas.2217011120

**Published:** 2023-02-22

**Authors:** Taisuke Yoneda, Kenji Hayashi, Yumiko Yoshimura

**Affiliations:** ^a^Division of Visual Information Processing, National Institute for Physiological Sciences, Okazaki, Aichi 444-8585, Japan; ^b^School of Life Science, SOKENDAI (The Graduate University for Advanced Studies), Okazaki, Aichi 444-8585, Japan; ^c^Graduate School of Medicine, Nagoya University, Nagoya, Aichi 466-8550, Japan

**Keywords:** subplate neuron, ocular dominance plasticity, visual cortex, layer 6b

## Abstract

Subplate neurons are early-born cortical neurons that transiently form neural circuits during perinatal development and guide cortical maturation. Thereafter, most subplate neurons undergo cell death, while some survive and renew their target areas for synaptic connections. However, the functional properties of the surviving subplate neurons remain largely unknown. This study aimed to characterize the visual responses and experience-dependent functional plasticity of layer 6b (L6b) neurons, the remnants of subplate neurons, in the primary visual cortex (V1). Two-photon Ca^2+^ imaging was performed in V1 of awake juvenile mice. L6b neurons showed broader tunings for orientation, direction, and spatial frequency than did layer 2/3 (L2/3) and L6a neurons. In addition, L6b neurons showed lower matching of preferred orientation between the left and right eyes compared with other layers. Post hoc 3D immunohistochemistry confirmed that the majority of recorded L6b neurons expressed connective tissue growth factor (CTGF), a subplate neuron marker. Moreover, chronic two-photon imaging showed that L6b neurons exhibited ocular dominance (OD) plasticity by monocular deprivation during critical periods. The OD shift to the open eye depended on the response strength to the stimulation of the eye to be deprived before starting monocular deprivation. There were no significant differences in visual response selectivity prior to monocular deprivation between the OD changed and unchanged neuron groups, suggesting that OD plasticity can occur in L6b neurons showing any response features. In conclusion, our results provide strong evidence that surviving subplate neurons exhibit sensory responses and experience-dependent plasticity at a relatively late stage of cortical development.

## Significance

During early development of the mammalian cortex, subplate neurons guide neuronal circuit formation. Most of them undergo cell death shortly after birth, while some survive and build new neural circuits. However, the function of the surviving subplate neurons remains largely unknown. This study found that surviving subplate neurons showed clear responses to visual stimuli with broad tunings in the primary visual cortex of juvenile mice. Furthermore, subplate neurons exhibited visual experience-dependent functional plasticity during critical periods, when visual inputs can effectively modify cortical function. These results suggest that surviving subplate neurons are involved in the experience-dependent maturation of cortical functions and information processing in the mature cortex.

The mammalian cerebral cortex consists of six layers, with distinct roles in information processing (1, 2). At the bottom of the neocortex, on the boundary between the gray matter and white matter, there is a thin sheet of neurons called layer 6b (L6b) (3). Layer 6b neurons are thought to be remnants of subplate neurons based on their location and cell-type marker expression (4). During prenatal and early postnatal periods, subplate neurons form transient neuronal circuits that play key roles in cortical maturation (5–7). In the embryonic cortex, subplate neurons form short-lived synapses with early immature neurons to regulate radial migration (8). During perinatal development, subplate neurons transiently receive inputs from ingrowing thalamic axons and innervate layer 4 (L4) to guide thalamic inputs to the eventual target, L4 (5, 6). Thus, the circuits formed by subplate neurons at the perinatal developmental stage are essential to establish basic neuronal circuits before starting experience-dependent refinements (5–7). Subsequently, subplate neurons largely disappear due to programmed cell death, but some survive and reside in L6b (5, 6). In the adult cortex, L6b neurons form neuronal circuits with local and long-distance neurons, which are different from those formed during early development (9–12). Therefore, surviving subplate neurons may acquire a role in information processing after remodeling of neuronal connections. A recent study using three-photon Ca^2+^ imaging demonstrated that L6b neurons show visual responses with broad orientation/direction tuning in the adult mouse primary visual cortex (V1) (13). However, comparable evidence for L6b response properties with other layer neurons in V1 is lacking (14–20). Moreover, L6b neurons have diverse morphology and molecular expression (21–24). Neurons born during subplate neurogenesis show the different expression patterns of subplate markers in postnatal L6b (4). However, the response properties in each subtype of L6b neurons remain unknown.

The sensory responsiveness of cortical neurons is considerably refined by sensory experience relatively late in development, referred to as the critical period (25, 26). Previous studies have demonstrated that sensory activities before the onset of the critical period affect the arrangement of subplate neuron neurites in the barrel cortex and local subplate circuits in the auditory cortex (27, 28). However, there is no direct evidence that the sensory responses of surviving subplate neurons are modified by sensory experience during the critical period. If experience-dependent plasticity occurs in subplate neuron responses, they will contribute to the experience-dependent development of sensory functions and possibly to the functions in the mature cortex. Ocular dominance (OD) plasticity in V1 is a canonical model used to examine experience-dependent refinement of sensory responses (25, 26, 29, 30). If one eye is occluded for several days during the critical period, neurons in V1 lose their response to the deprived eye. OD plasticity is robustly preserved across species and cell types. Therefore, OD plasticity is suitable for evaluating experience-dependent plasticity in L6b neurons.

This study aimed to characterize the visual responses and OD plasticity of L6b neurons in V1. Toward this goal, two-photon Ca^2+^ imaging was performed in awake juvenile mice, followed by 3D immunohistochemistry with a subplate neuronal marker, connective tissue growth factor (CTGF) (4, 31). L6b neurons showed broader tuning to visual stimuli and lower binocular matching of orientation preference than did layer 2/3 (L2/3) and L6a neurons. Chronic two-photon imaging revealed significant OD plasticity in individual L6b neurons during the critical period. Our results provide strong evidence that L6b neurons, presumed to be subplate neuron remnants, exhibit sensory responses and experience-dependent functional plasticity at a relatively late stage of cortical development.

## Results

### Broadly Tuned Receptive Field Properties of Layer 6b Neurons in the Developing Mouse V1.

**Fig. 1.**
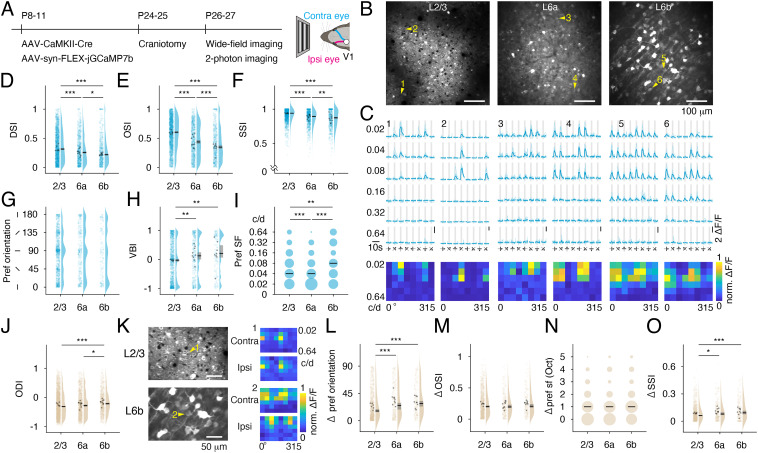
Receptive field properties of L2/3, L6a, and L6b neurons in V1. (*A*, *Left*) Experimental time course. (*Right*) Scheme of two-photon imaging of visual responses to contralateral (Contra) and ipsilateral (Ipsi) eye stimuli. White circle shows the recording V1 hemisphere. (*B*) Examples of two-photon field of views (FOVs) in L2/3, L6a, and L6b. Yellow arrowheads indicate the location of neurons whose activity is shown in *C*. (*C*, *Top*) Individual (light blue) and average (dark blue) calcium responses to grating stimuli of eight directions and six spatial frequencies delivered to the contralateral eye. Gray rectangles represent the stimulation period. Bottom: Heatmaps of average responses during the stimulation period. (*D*–*H*) The distribution of direction selectivity index (DSI, *D*), orientation selectivity index (OSI, *E*), spatial frequency selectivity index (SSI, *F*), preferred orientation (*G*), and vertical bias index (VBI, *H*) of contralateral eye responses in each layer. Pale-colored and black dots show the values from individual neurons and the mean data of individual mice, respectively. Black lines and gray squares on the kernel density estimation indicate the median and 95% CI. (*I*) The distribution of preferred spatial frequency. The size of the circles shows the density of each spatial frequency. Black lines represent the median. (*J*) Distribution of ocular dominance index (ODI). (*K*, *Left*) Examples of two-photon FOVs in L2/3 and L6b. (*Right*) Heatmaps of average responses to contralateral (Contra) and ipsilateral eye stimulation (Ipsi) from the neurons shown in the arrowheads in the *Left* panels. (*L*–*O*) Difference in preferred orientation (Δpref orientation, *L*), OSI (ΔOSI, *M*), preferred spatial frequency (Δpref sf, *N*), and SSI (ΔSSI, *O*) of the two eyes. The number of animals and cells are as follows: n = 6 mice (L2/3), nine mice (L6a), and nine mice (L6b); (*D*–*F*): n = 1,868 cells (L2/3), n = 847 (L6a), and n = 590 (L6b); (*G* and *H*): n = 1,086 (L2/3), n = 356 (L6a), and n = 156 (L6b); (*I*): n = 1,261 (L2/3), n = 510 (L6a), and n = 313 (L6b); (*J*): n = 2,052 cells (L2/3), n = 1,031 cells (L6a), and n = 686 cells (L6b); (*L*–*O*): n = 819 (L2/3), n = 243 (L6a), and n = 191 (L6b). Permutation test (*D*–*F*, *H*, *J*, *L*, *M*, and *O*) and Mann–Whitney *U* test test with Holm–Bonferroni method (*I* and *N*) are performed (*P < 0.05, **P < 0.01, ***P < 0.001).

We investigated the visual response properties and experience-dependent plasticity in L6b neurons of developing mouse V1. Two-photon Ca^2+^ imaging with jGCaMP7b expressed virally in excitatory neurons of head-fixed awake mice was performed on postnatal days 26 to 27 (P26–27) (Fig. 1*A*). To functionally determine the binocular area of V1, wide-field Ca^2+^ imaging was performed before two-photon imaging (Fig. 1*A* and *SI Appendix*, Fig. S1). The area responsive to visual stimuli in the binocular visual field was defined as the binocular area (*SI Appendix*, Fig. S1 *A* and *B*). In the mice subset that underwent Ca^2+^ imaging, the callosal projection zone was labeled with a retrograde tracer to anatomically confirm the binocular area (*SI Appendix*, Fig. S1*C*). Based on the vascular pattern of the brain surface, we selected a field of view (FOV) in the V1 binocular area and recorded visual responses from L2/3, L6a, and L6b neurons using two-photon Ca^2+^ imaging (Fig. 1*B*). Examples of contralateral (Contra) eye responses in L2/3, L6a, and L6b neurons are shown in Fig. 1*C*. Receptive field properties for individual neurons were estimated from the responses to drifting sinusoidal gratings of each combination of eight directions and six spatial frequencies. We explored the receptive field properties of the contralateral eye responses at L2/3, L6a, and L6b. The direction selectivity index (DSI), orientation selectivity index (OSI), and spatial frequency selectivity index (SSI) calculated from contralateral eye responses were significantly lower in L6b neurons than in L2/3 or L6a neurons (Fig. 1 *D*–*F*). Similar to the contralateral eye responses, ipsilateral (Ipsi) eye responses in L6b neurons showed lower selectivity for all tested visual features (*SI Appendix*, Fig. S2 *A*–*D*). These results suggest that L6b neurons have broader tuning properties to visual stimuli than do L2/3 and L6a neurons.

The distribution of the preferred orientations of the contralateral eye responses in each layer is shown in Fig. 1*G*. The vertical biased index (VBI) in L6b was more positive than that in L2/3 and similar to that in L6a, demonstrating that preferred orientation distribution in L6 neurons is biased toward vertical orientation (Fig. 1 *G* and *H*). The preferred spatial frequencies of contralateral eye responses were significantly higher in L6b than in L2/3 or L6a (Fig. 1*I*). However, ipsilateral eye responses showed no significant difference in the VBI between L6b and L2/3 or L6a (*SI Appendix*, Fig. S2 *E* and *F*). In addition, the preferred spatial frequencies of ipsilateral eye responses in L6b were lower than those in L2/3 but similar to those in L6a (*SI Appendix*, Fig. S2*G*). Previous studies in L2/3 demonstrated that contralateral eye responses tuned to high spatial frequency preferentially showed vertical orientation bias (32, 33). This preference was not observed in ipsilateral eye responses (32). Thus, these L2/3 response properties may affect L6b responses with vertical orientation bias and high spatial frequency.

### Weaker Binocular Matching of Layer 6b Neurons.

Next, we examined the binocular properties of the L6b neurons. On average, the ocular dominance index (ODI) was negative in all the tested layers, indicating contralateral eye dominance (Fig. 1*J*) (34). The ODI in L6b neurons was closer to 0 than that in L2/3 and L6a neurons, demonstrating that L6b neurons showed relatively high binocularity (Fig. 1*J*). Matching of visual responses from each eye in binocular neurons was examined (Fig. 1 *K*–*O*). The preferred orientation in L6b neurons was more binocularly mismatched than that in L2/3 neurons (Fig. 1*L*). The binocular difference in the OSI was comparable across all tested layers (Fig. 1*M*). The difference in the preferred spatial frequencies of both eyes were similar in all layers (Fig. 1*N*), although the preference seemed different between the contralateral and ipsilateral eye responses when monocular neurons were included (Fig. 1*I* and *SI Appendix*, Fig. S2*G*). The binocular difference in SSI was slightly, but significantly, larger in L6b than in L2/3 (Fig. 1*O*). These results indicate that L6b neurons have weaker binocular matching than do other cortical neurons.

### Visual Response Properties in Layer 6b Neuron Subtypes Identified with Post Hoc 3D Immunostaining.

**Fig. 2.**
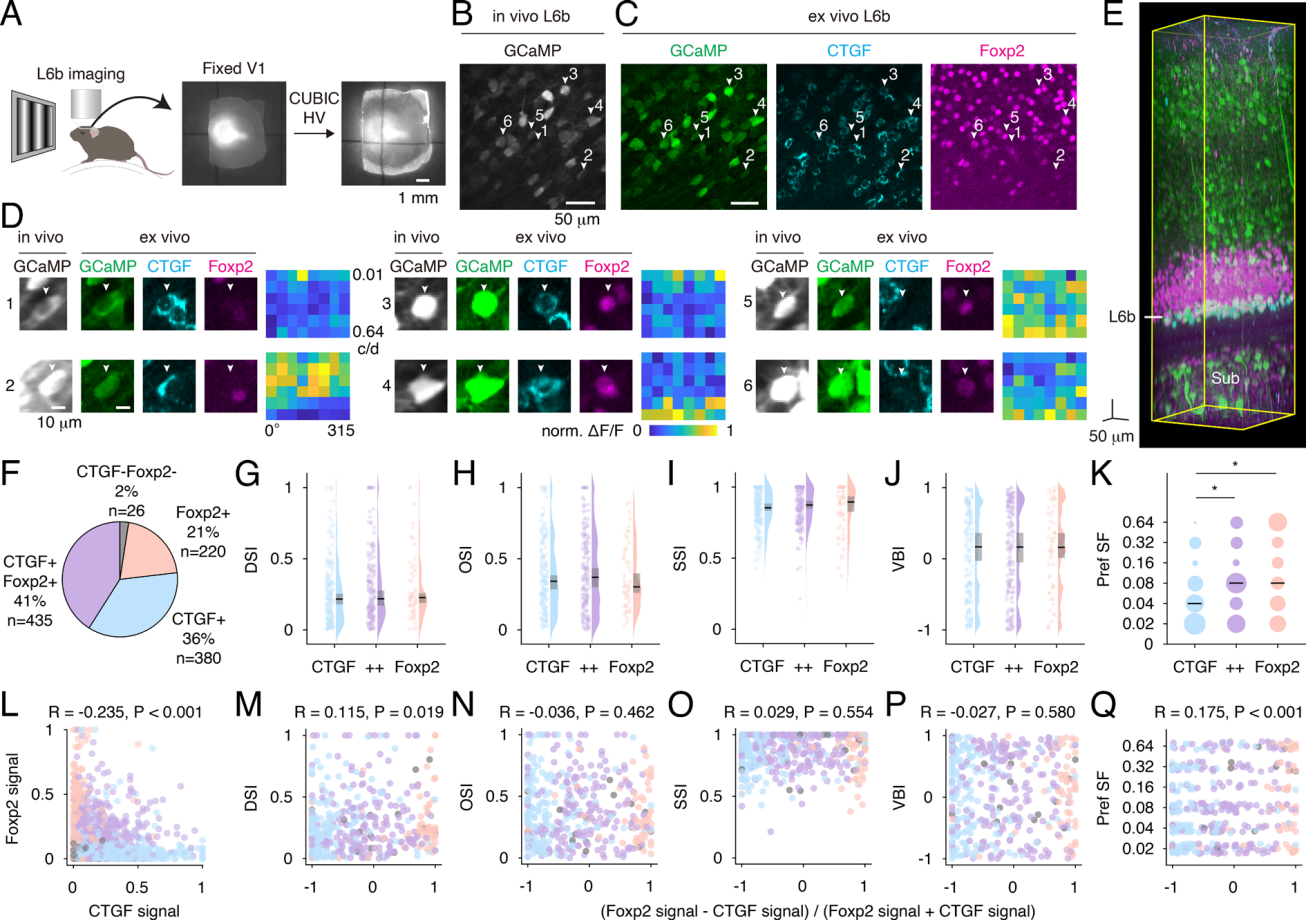
Receptive field properties of L6b subtypes. (*A*) A fixed brain before (*Left*) and after (*Right*) CUBIC-HV. (*B* and *C*) GCaMP-labeled L6b neurons from in vivo two-photon imaging (*B*) and the same areas from a fixed brain after CUBIC-HV (*C*). White arrowheads indicate the CTGF (+), CTGF (+) Foxp2 (+), and Foxp2(+) neurons whose activity is shown in *D*. (*D*) Neuron images and heatmaps of the average responses of L6b subtypes. Cells 1 and 2 are CTGF (+), cells 3 and 4 are CTGF (+) Foxp2 (+), and cells 5 and 6 are Foxp2(+). (*E*) An example of a volumetric image of clearing V1 with CUBIC-HV. A horizontal white line on the left indicates the recording depth for L6b. Sub: Subiculum. (*F*) Proportions of CTGF (+) Foxp2 (+) (purple), CTGF (+) (blue), Foxp2(+) (pink), and CTGF (-) Foxp2 (-) (gray). (*G*–*J*) The distribution of direction selectivity index (DSI, *G*), orientation selectivity index (OSI, *H*), spatial frequency selectivity index (SSI, *I*), and vertical bias index (VBI, *J*) of contralateral eye responses in each subtype. Pale-colored and black dots show the values from individual neurons and the mean data of individual mice, respectively. Black lines and gray squares on the kernel density estimation indicate the median and 95% CI. (*K*) Preferred spatial frequency distribution. The size of the circles shows the density of each spatial frequency. Black lines represent the median. (*L*) Relationship between CTGF and Foxp2 immunoreactivity among in vivo recorded L6b neurons. The color of each circle shows a cell type in *F*. (*M*–*Q*) Relationship between Foxp2/CTGF signal index and visual selectivity (DSI, OSI, SSI, VBI, and Pref SF). The number of animals and visually responsive cells are as follows: n = 8 mice in each subtype; (*G*–*K*, *M*–*Q*): n = 175 cells [CTGF (+)], n = 175 [CTGF (+) Foxp2 (+)], and n = 56 [Foxp2(+)]. Permutation test (*G*–*J*) and Mann–Whitney *U* test test with Holm–Bonferroni method (*K*) are performed (*P < 0.05).

After recording, the brain was fixed, and tissue clearing was performed using the CUBIC-HV method (Fig. 2 *A*–*C*) (35). A previous study demonstrates that neurons born during the subplate neurogenesis express CTGF in the postnatal cortex (4). To identify L6b neuron subtypes, 3D immunostaining for CTGF (a marker of subplate neurons) and forkhead box P2 (Foxp2, a marker of cortico-thalamic neurons) was conducted. CTGF-positive signals were distributed in L6b (Fig. 2 *C*–*E* and *SI Appendix*, Fig. S3). L6b neurons that underwent in vivo imaging were assigned to the cleared brain (Fig. 2 *B*–*D*). Among the excitatory neurons expressing GCaMP in L6b, 36% CTGF-single-positive, 21% Foxp2-single-positive, and 41% CTGF- and Foxp2-double-positive neurons were detected (Fig. 2*F*). Thus, most of the recorded L6b neurons expressed CTGF, demonstrating they are subplate neurons (4). This trend was common in male and female mice [CTGF-single positive (35% males and 37% females), Foxp2-single positive (19% males and 23% females), CTGF- and Foxp2-double positive (43% males and 38% females), *P* > 0.05]. CTGF-positive neurons showed bipolar or multipolar cell-like morphology, which probably corresponds to the L6b/subplate neuron subtypes identified by biocytin staining (*SI Appendix*, Fig. S4) (23).

The visual response properties’ comparison among CTGF-single-positive, Foxp2-single -positive, and double-positive neurons showed no significant differences in DSI, OSI, and SSI among the three L6b neuron groups (Fig. 2 *G*–*I*). As previously mentioned when different cortical layers were compared, VBI and preferred spatial frequency were estimated using orientation and spatial frequency selective neurons, respectively (Fig. 1 *H* and *I*). Similar trends were shown using visual responsive cells in each layer (*SI Appendix*, Fig. S5). Thus, visual responsive neurons were used to compare these features among L6b neuron subtypes. There was no significant difference in VBI among the three L6b subtypes (Fig. 2*J*). However, the preferred spatial frequencies in CTGF-single-positive neurons were lower than those in the other two groups (Fig. 2*K*). The signal intensities of CTGF and Foxp2 in L6b neurons were observed to be negatively correlated (Fig. 2*L*). The Foxp2/CTGF signal index was calculated to quantify the signal intensity of Foxp2 and CTGF in individual L6b neurons, and visual selectivity indices were plotted against the signal index. The DSI was weakly correlated with the Foxp2/CTGF signal index (Fig. 2*M*), although no significant difference in the DSI among the three grouped L6b subtypes were detected (Fig. 2*G*). In addition, no significant correlation existed between the Foxp2/CTGF signal index and the OSI, SSI, or VBI (Fig. 2 *N*–*P*). However, the Foxp2/CTGF signal indices were significantly correlated with the preferred spatial frequencies (Fig. 2*Q*), consistent with the comparison among the three L6b subtypes (Fig. 2*K*). These results suggest that each heterogeneous cell population in L6b may exhibit different visual response properties.

### Experience-Dependent Plasticity of Layer 6b Neurons.

**Fig. 3.**
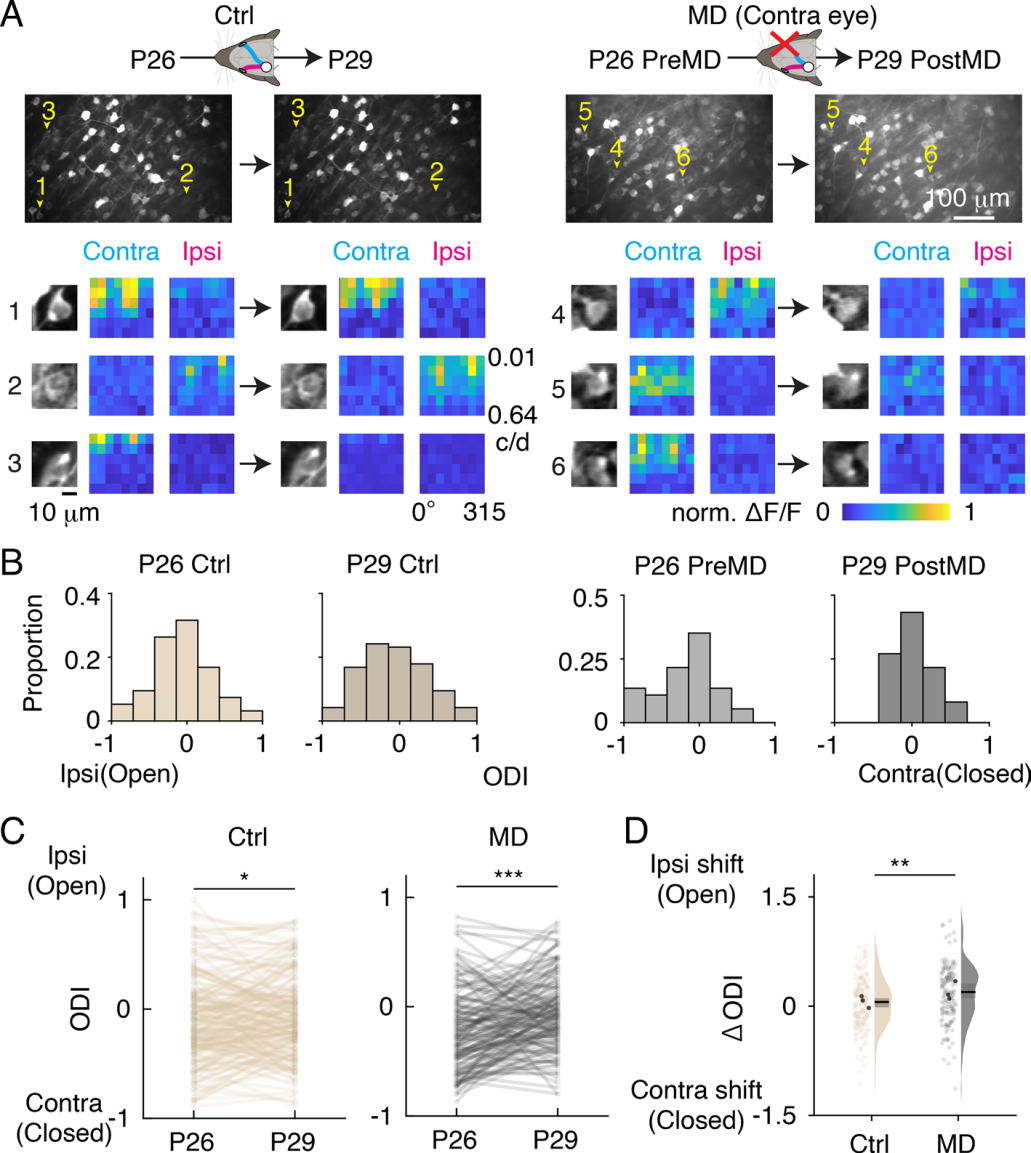
Ocular dominance plasticity of L6b neurons. (*A*) Chronic two-photon imaging in control (Ctrl, *Left*) and monocular-deprived (MD) mice (*Right*). (*Top*) Examples of FOVs. Yellow arrowheads indicate the location of neurons shown at the bottom. (*Bottom*) Neuron images and heatmaps of the average responses of chronically recorded L6b neurons. Cells 1, 2, and 4 exhibit stable ocular dominance. Cells 3, 5, and 6 show depression of contralateral eye responses. (*B*) Ocular dominance histogram of a control mouse (*Left*) and an MD mouse (*Right*) in each imaging session as shown in *A*. (*C*) Ocular dominance index (ODI) in the control (n = 216 cells from 3 mice) and MD mice (n = 156 cells from 3 mice). ODI for the same neuron between two imaging sessions is connected with lines. (*D*) Distribution of the difference in ODI (ΔODI) between P26 and P29. Pale and black circles show the values from individual neurons and the mean data of individual mice, respectively. Black lines and gray squares on the kernel density estimation indicate the median and 95% CI. Wilcoxon signed-rank test (*C*) and permutation test (*D*) are performed (*P < 0.05, **P < 0.01, ***P < 0.001).

To examine whether L6b cells exhibit experience-dependent plasticity, we repeated recordings of visual responses from the same L6b neurons before (at P26) and after monocular deprivation (at P29) using two-photon Ca^2+^ imaging (Fig. 3*A* and *SI Appendix*, Fig. S6). In mice subset that underwent the chronic two-photon imaging, chronic wide-field Ca^2+^ imaging confirmed that monocular deprivation for 3 d elicited a significant OD shift in V1 population activities (*SI Appendix*, Fig. S7). The chronic two-photon Ca^2+^ imaging demonstrated that the OD of L6b neurons was significantly shifted to the ipsilateral open eye after monocular deprivation (Fig. 3 *B* and *C*). The ipsilateral shift also occurred in control mice without monocular deprivation, consistent with a previous study in L2/3 neurons (36). However, the OD changes were more prominent in monocular-deprived (MD) mice. To quantify OD changes in individual L6b neurons, we evaluated the difference in ODI (ΔODI) between P26 and P29. The ΔODIs of monocular-deprived mice were distributed in more positive values than those of control mice, demonstrating a strong OD shift to the open ipsilateral eye after monocular deprivation (Fig.  3*D*). The OD shift in deprived mice was caused by depression in response to eye closure (*SI Appendix*, Fig. S8). These results suggest that L6b neurons exhibit experience-dependent plasticity during postnatal development.

### Visual Response Properties of the Layer 6b Neurons with Significant Plasticity.

**Fig. 4.**
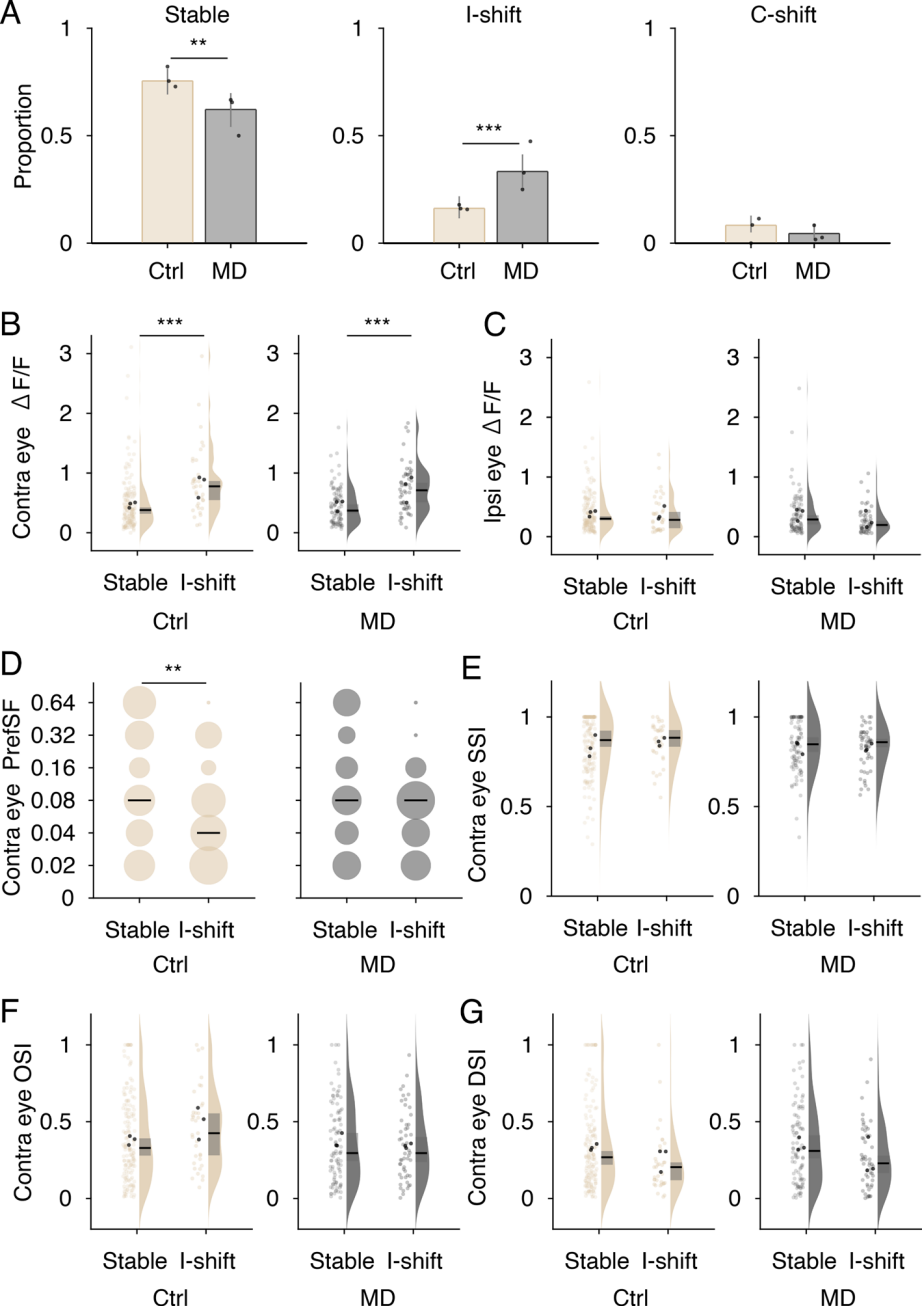
Characterization of stable and plastic L6b neurons in control (Ctrl) and monocular-deprived (MD) mice. (*A*) The proportion of neurons with unchanged ocular dominance index (Stable), a shift to the ipsilateral eye (I-shift), and a shift to the contralateral eye (C-shift) in control and MD mice (n = 3 mice each). In MD mice, ipsilateral eye was the nondeprived eye, and contralateral eye was the deprived eye. Black circles indicate the mean data of individual mice. (Ctrl, n = 163 cells (Stable), n = 35 (I-shift), n = 18 (C-shift); MD, n = 97 (Stable), n = 52 (I-shift), n = 7 (C-shift)). Error bars represent the 95% CI. (*B* and *C*) The distribution of response amplitude to the contralateral eye stimulation (Contra eye ΔF/F) and that to the ipsilateral eye stimulation (Ipsi eye ΔF/F) in stable and I-shift groups. Pale-colored and black dots show the values of individual neurons and the mean data of individual mice, respectively. Black lines and gray squares on the kernel density estimation indicate the median and 95% CI. (*D*) Distribution of preferred spatial frequencies of contralateral eye responses. The size of the circles indicates the density. Black lines represent median values. (*E*–*G*) Distribution of spatial frequency selectivity index (SSI, *E*), orientation selectivity index (OSI, *F*), and direction selectivity index (DSI, *G*) of contralateral eye. Data in *B*–*G* are obtained at P26 (before MD). Fisher's exact test (*A*), permutation test (*B*, *C*, and *E*–*G*), and Wilcoxon signed-rank test (*D*) are performed (**P < 0.01, ***P < 0.001).

Finally, we explored whether the OD plasticity of L6b neurons is associated with visual response properties prior to monocular deprivation. Using a bootstrap method, individual neurons were classified into three groups according to the OD changes during P26–P29: stable OD and OD shift to the ipsilateral eye (ipsilateral shift) and the contralateral eye (contralateral shift) (*SI Appendix*, Fig. S9). The proportion of neurons with stable OD was significantly lower and that with ipsilateral shift (open eye shift) was higher in monocular-deprived mice than in the control mice (Fig. 4*A*). Less than 10% of the analyzed neurons showed a contralateral shift in control and monocular-deprived mice. To characterize the neurons susceptible to OD changes, the visual response properties at P26 (before monocular deprivation) were compared between the stable OD and ipsilateral-shift groups in control or deprived mice. The visual responses to contralateral eye stimuli were larger in the ipsilateral-shift group than in the stable OD group in both control and monocular-deprived mice (Fig. 4*B*). In contrast, the response strength to ipsilateral eye stimuli was comparable between the two groups (Fig. 4*C*). There was no significant difference in the preferred spatial frequencies between the stable OD and ipsilateral-shift groups in monocular-deprived mice (Fig. 4*D*). However, the frequencies were significantly lower in the ipsilateral-shift group than that in the stable OD group in the control mice (Fig. 4*D*). There were no differences in SSI, OSI, or DSI between the two groups in the control and deprived mice (Fig. 4 *E*–*G*). These data suggest that L6b neurons with strong responses to contralateral eye stimulation exhibit an OD shift to the ipsilateral eye after monocular deprivation independent of their receptive field properties.

## Discussion

This study focused on the visual response properties and functional plasticity in L6b neurons, surviving subplate neurons, in V1 at a relatively late developmental stage. Two-photon Ca^2+^ imaging was applied to L6b, the deepest part of the cortex, in the V1 of awake mice during the critical period. The results showed that L6b neurons were broadly tuned to orientation and direction in juvenile mouse V1, consistent with a previous study in adult mice (13). In addition, there was broader spatial frequency tuning and lower binocular matching of orientation preference in L6b neurons than in L2/3 and L6a neurons. Post hoc 3D immunohistochemistry confirmed that the majority of recorded L6b neurons express a subplate marker, CTGF. Thus, it is likely that the L6b neurons analyzed here are remnants of subplate neurons. Chronic two-photon imaging of the same neurons before and after monocular deprivation revealed that L6b neurons showed OD plasticity. This is a description of experience-dependent functional plasticity in surviving subplate neurons during relatively late postnatal development.

### Receptive Field Properties of Subplate Neurons and Their Implications.

Surviving subplate neurons are incorporated into the cortical circuits. In the adult cortex, L6b neurons provide feedback modulatory input to the higher order thalamus (9, 11). Adult L6b neurons also project to higher order cortical areas, such as the cingulate, retrosplenial, and orbital cortices (9). The current study found that L6b neurons were more broadly tuned to visual stimulus features than were L2/3 and L6a neurons. The trends are likely to be kept in adult V1 because a previous study reported broad tunings for orientation and direction in adult L6b (13). Thus, L6b neurons, including subplate marker-positive neurons, can provide relatively rough visual information to the recipient areas. In addition, the recipient areas can receive approximate visual information from either the left or right eye because of high binocularity and low binocular matching in L6b neurons, although binocular matching may be improved until adulthood, similar to other cortical neurons (37, 38).

L6b has a diverse excitatory neuronal population (21–24). Indeed, CTGF and/or Foxp2-positive neurons were found in L6b. A previous study indicates that *CTGF*-mRNA expression is constant from P8 to adult in L6b of the mouse somatosensory cortex (21). The proportion of CTGF-positive neurons among L6b neurons in the juvenile visual cortex (P26–27, Fig. 2*F*) is comparable to that in the P8 somatosensory cortex (4), suggesting CTGF as a stable subplate neuron marker after the perinatal stage. Importantly, CTGF-single-positive neurons showed a preference to lower spatial frequencies. Therefore, individual L6b neuron subtypes may have a distinct role in visual information processing.

A previous study has demonstrated that L6b neurons, rather than ongoing sensory processing, contribute to increasing activity in the initial phase of repetitive whisker stimulation in the higher-order thalamus (11). L6b neurons in the entorhinal cortex contribute to spatial information processing (12). L6b neurons are the only cortical neurons sensitive to orexin, which regulates wakefulness (39). Therefore, subplate neurons in the adult cortex may exert modulatory impacts on their target areas depending on behavioral contexts or brain states.

Although L6b transiently innervates the first-order thalamus during the perinatal period, L6a neurons preferentially project to the thalamus in adults (6, 9, 11, 19, 40). Cortico-thalamic neurons in the L6a exhibit sharper orientation selectivity than callosal projection neurons residing in the same layer (16, 20). In our study, the selectivity for orientation, direction, and spatial frequency was sharper in L6a than that in L6b. This indicates that the first-order thalamus can receive feedback modulatory inputs that represent precise visual-stimulus features in adults.

### Experience-Dependent Plasticity for Remnants of Subplate Neurons.

Ablation of subplate neurons during early development prevents the maturation of GABAergic transmission, leading to setting errors in the direction of OD plasticity during the subsequent critical period in cat V1 (41). We found that subplate neurons themselves exhibit OD plasticity during the critical period, similar to neurons in other layers of V1 (34, 42, 43). OD plasticity derived from deprived-eye depression after 3 d of monocular deprivation is a common feature of L6b neurons and other V1 neurons (44–47). Thus, the function of subplate neurons at this developmental phase can be modified in coordination with other visual cortical neurons in an experience-dependent manner, possibly to adapt to the external visual environment.

A previous study demonstrated that the OD of pyramidal cells in the upper part of L6 is unchanged after monocular deprivation during the sensitive period (43). Thus, plasticity in neurons within L6 may differ depending on cell type. In this study, recording was limited to excitatory neurons, and most recorded neurons in L6b were CTGF-positive subtypes. However, excitatory neurons in the L6b are heterogeneous (21–24). Thus, it is possible that the effect of visual manipulation is determined by the cell type. Further studies are required to confirm this hypothesis.

*SI Appendix*, Fig. S10 shows the possible neural circuits underlying OD plasticity in L6b. Given that thalamocortical projections from the lateral geniculate nucleus (visual thalamus) to the subplate layer disappear before the sensitive period for OD plasticity begins, the thalamocortical pathway cannot be involved in the L6b plasticity. Subplate neurons in the adult cortex receive local inputs from L2/3 and intratelencephalic neurons in deep cortical layers, and a few inputs from L5 pyramidal tract and L6 corticothalamic neurons (10). They also receive long-range inputs from the contralateral and higher cortices (10). The visual responses to the closed contralateral eye were depressed following monocular deprivation. Given that the callosal inputs enhance visual responses to the ipsilateral eye in V1 (48), their contribution is unlikely. A previous study indicates that L2/3 neurons show OD plasticity but not L5 intratelencephalic neurons and L6 neurons (43). Therefore, OD plasticity in L6b may reflect the plastic changes in L2/3 and be based on long-term depression at L6b synapses originating from L2/3, L5, and L6. Indeed, long-term depression is induced at synapses on subplate neurons in the rat visual cortex (49).

Our chronic imaging of visual responses revealed that the OD shift to the ipsilateral open eye after monocular deprivation occurred in L6b depending on the response strength to contralateral eye stimuli prior to deprivation. In addition, there were no significant differences in visual response selectivity prior to monocular deprivation between the OD changed and unchanged neuron groups, suggesting that OD plasticity can occur in L6b neurons with any response features. L6b neurons showed a dynamic change in ODs for 3 d during the critical period in both mice with and without monocular deprivation. However, the proportion of neurons with OD shift to the ipsilateral eyes in monocularly deprived mice was double that in normal mice, demonstrating that OD plasticity occurs by monocular deprivation.

In normal mice without monocular deprivation, some L6b neurons tuned to a relatively low spatial frequency; these neurons preferentially showed OD shift to the ipsilateral eye for 3 d. Meanwhile, other L6b neurons tuned to a relatively high spatial frequency; OD was unchanged in these neurons. A previous study conducted with chronic two-photon Ca^2+^ imaging from the onset to the end of the critical period demonstrated that in L2/3, binocular neurons with a low spatial frequency preference and weak orientation tuning are converted into monocular neurons, and monocular neurons tuned to high spatial frequency and with sharp orientation selectivity convert into binocular neurons during normal development (36). Thus, the ODs of L6b neurons may convert depending on the initial visual response selectivity in the process of normal development, although longer tracking of L6b responses will be necessary to prove this.

At the early developmental stage, subplate neurons guide the establishment of thalamocortical connections, depending on spontaneous and sensory activities (5, 6). During early development, the arrangement of subplate neuron neurites in the barrel cortex and local subplate circuits in the auditory cortex are altered by sensory deprivation (27, 28). We found that, at a later developmental stage, sensory responses in L6b neurons were modified in an experience-dependent manner. A limitation of this study is that the functional role of the plasticity of L6b neurons was not elucidated. A recent study demonstrated that L6b neurons in the entorhinal cortex were involved in spatial memory in adult mice (12). Thus, the plasticity of individual L6b neurons may contribute to experience- and learning-dependent modification of cortical function.

The pathogenetic increase in subplate remnants and abnormality of time specificity of neuronal activities presumably derived from subplate neurons are related to neurological and psychiatric disorders in humans (6). This study provides an additional aspect of L6b functions. Further investigations into the functional roles or perturbation of L6b plasticity may shed light into the mechanisms by which L6b neurons contribute to cortical development and function and the overall pathophysiology of developmental or memory disorder.

## Materials and Methods

### Mice.

This study was approved by the Experimental Animal Committee of the National Institute for Physiological Sciences. Male and female wild-type C57BL/6J mice were used (Japan SLC). Female ICR (Japan SLC) mice were used as the foster parents.

### AAV Construction.

pGP-AAV-syn-FLEX-jGCaMP7b-WPRE was a gift from Douglas Kim & GENIE Project (Addgene viral prep # 104493-AAV1; RRID:Addgene_104493). AAVDJ-CaMKII-Cre was prepared using pENN.AAV.CamKII 0.4.Cre.SV40 and pENN.AAV.CamKII.HI.GFP-Cre.WPRE.SV40. pENN.AAV.CamKII 0.4.Cre.SV40 was a gift from James M. Wilson (Addgene plasmid # 105558; RRID:Addgene_105558). pENN.AAV.CamKII.HI.GFP-Cre.WPRE.SV40 was also a gift from James M. Wilson (Addgene plasmid # 105551; 105551; RRID:Addgene_105551).

### AAV Injection.

Virus injection, craniotomy, and tracer injection were performed as previously described (50). C57BL/6J mice at P7–9 were fostered in ICR mice. Mice (P8–11) were anesthetized with isoflurane (5% for induction and 1 to 3% for maintenance) and maintained at 37 °C using a heating pad. After the mice were placed in the stereotaxic instrument, the scalps were incised to expose the skull surfaces. The small hole was made at the coordinates of 2.6 mm lateral and 1.6 mm anterior to lambda of the skull. Glass micropipettes with a 30 to 40-μm tip diameter were filled with a mixture solution of AAVDJ-CaMKII-Cre (5.0 to 7.5 × 10^−12^ GC/mL) and AAV1-syn-FLEX-jGCaMP7b (3.8 × 10^−12^ GC/mL). The glass micropipettes were tilted at an angle of 30° to the horizontal plane and were inserted 1.4 mm. The micropipettes were located ~700 μm below the cortical surface at the center of the cranial window. A virus solution (0.4 μL) was injected (Nanoject II, Drummond) for 20 min. The surgical field was carefully washed with a saline solution. After retraction of the glass micropipette, the scalps were sutured. The mice were recovered from anesthesia on a heating pad.

### Craniotomy and Tracer Injection.

The mice reaching P24–25 were anesthetized with isoflurane (5% for induction and 1 to 3% for maintenance). Mice were intraperitoneally administered carprofen (0.03 mL 5 mg/mL, Zoetis) to reduce inflammation, and glycerol (0.1 mL) to avoid cerebral edema. Mice were placed on the stereotaxic instrument and maintained at 37 °C using a heating pad. The eyes were coated with an ointment (ofloxacin, TOA Pharmaceuticals) to prevent dehydration. After removing the parietal scalp, the exposed skull was treated with an etching agent (Super-Bond C&B Green Activator; Sun Medical). Craniotomy was performed to expose V1 of the left hemisphere (3.0 mm lateral to lambda). The dura was exposed by removing the skull (3 mm in diameter) and carefully excised with forceps. The cut ends of the dura were washed with chilled 0.9% saline or 0.4% aminoglycosides, amikacin, and antibiotics in saline until the bleeding stopped. To avoid further bleeding, small pieces of the Avitene™ microfibrillar hemostat (Davol) were placed on the cut ends of the dura.

Window glass was prepared by gluing two disks of glass (#1 thickness, 3 mm and 4 mm in diameter) using a UV curing resin (NOA61, Norland Products). The window glass was directly applied over the craniotomy site with a smaller disk. Instant glue (Aron Alpha A, Sankyo) was applied to the rim of the window glass to create a seal between the glass and skull. In a subset of mice, cholera toxin B subunit (CTB) 405 or CTB555 (0.4 μL of 10 μg/μL in phosphate-buffered saline [PBS]) was injected into the right V1 to label the lateral border of the binocular region of the left V1 (51). A custom-made headplate was then fixed to the skull using dental resin cement (Super-Bond C&B, Sun Medical). The junction between the head plate and skull was covered with carbon-mixed resin (UNIFAST II, GC Corporation) for light shielding. The mice were placed on a heating pad and monitored until recovery. After surgery, carprofen (0.03 mL of 5 mg/mL) was administered for up to 1 d before two-photon imaging.

### Monocular Deprivation.

The mice at P26 were anesthetized with isoflurane (5% for induction and 1 to 3% for maintenance). The right eye was deprived of vision by using an eyelid suture. The sutured eyelids were treated with an antibacterial ointment (ofloxacin, TOA Pharmaceuticals). Lid closure was checked daily.

### Wide-Field Calcium Imaging.

For wide-field Ca^2+^ imaging, visual stimuli were generated using MATLAB (RRID: SCR_001622, MathWorks) with the Psychophysics Toolbox. A display (FS2333, EIZO) was placed 20 cm in front of each mouse. Visual stimuli were localized at −20° to 20° azimuth and in the upper visual field. As a result, segregated responses were observed in V1 and the anterior part of the higher visual areas (*SI Appendix*, Figs. S1 and S7). Sine-wave drifting gratings (100% contrast) using combinations of six SFs (0.02 to 0.64 c/d, in one-octave steps) at a fixed temporal frequency (2 Hz) and in the ventrally moving direction were alternately presented to the left eye and the right eye. Each stimulus was displayed 10 times for a 2-s duration, and the interstimulus interval (presenting a mean luminance gray) was 4 s. Wide-field Ca^2+^ imaging was performed in head-fixed awake mice at P26–27 with a custom stereotaxic instrument. The region including V1 was imaged at a 4-Hz frame rate using a Leica M165 FC stereomicroscope and a cooled CCD camera system (AQUACOSMOS with ORCA-ER camera, Hamamatsu Photonics). The excitation (Ex) and emission (Em) wavelengths were 450 to 490 nm and 500 to 550 nm, respectively. A subset of mice was repeatedly imaged at P29–30.

GCaMP fluorescence was averaged for each visual stimulus. The response magnitude in each pixel is defined as follows: $\Delta F/F=\left(F-F_{base}\right)/F_{base}$ , where F is the average fluorescence value during the stimulation period of 2 s, and $F_{\mathrm{base}}$ is that during the 2 s before each stimulus. The response maps of the contralateral and ipsilateral eyes were constructed from the signals of the preferred spatial frequency in each pixel. Regions of interest (ROIs, 500 × 500 µm) were set in the binocular area of the V1. In a subset of mice that underwent the wide-field imaging, the ROIs corresponded to FOVs of two-photon imaging. ODI is calculated as ΔF/F of the contralateral and ipsilateral eyes as follows: $\left({\Delta F/F}_{ipsi}-{\Delta F/F}_{cont}\right)/\left({\Delta F/F}_{ipsi}+{\Delta F/F}_{cont}\right)$ , where ${\Delta F/F}_{ipsi}$ and ${\Delta F/F}_{cont}$ are the peak responses of each eye.

### Two-Photon Imaging.

For two-photon Ca^2+^ imaging, visual stimuli were generated using PsychoPy. A gamma-corrected display (FS2333, EIZO) was placed 20 cm in front of each mouse. The display covered approximately 100° in azimuth and 70° in elevation of the visual space. Full-screen sine-wave drifting gratings (100% contrast, 100 cd/m^2^ mean luminance) using combinations of six SFs (0.02 to 0.64 c/d, in one-octave steps) in eight directions (0 to 315°, in 45° steps) at a fixed temporal frequency (2 Hz) were presented to the left eye and the right eye alternately. In addition to the 48 grating stimuli, a blank condition (full-screen mean luminance blank trials) and a condition in which the entire monitor flickered at 2 Hz (flash trials) were included. A total of 50 stimuli were presented in a pseudorandom order with 5 to 7 repeats for each imaging session. Each stimulus was displayed for four frames of two-photon imaging, and the interstimulus interval (mean luminance gray) lasted eight frames.

For awake mouse imaging, the heads of mice were fixed to a custom stereotaxic instrument. GCaMP fluorescence was acquired using a two-photon microscope (A1R MP, Nikon) equipped with a Mai Tai DeepSee laser and water immersion objective lens (25×, NA 1.10, MRD77220, Nikon). GCaMP was excited at 950 nm, and the fluorescence was routed to multi-alkali PMTs using a dichroic mirror (560 nm) and an emission filter (500 to 550 nm). Images were obtained using the Nikon NIS Elements software. A square region of V1 (512 × 512 pixels, pixel size = 0.99 μm) was recorded at 1.93 Hz. In all mice, in vivo imaging was performed in each layer at a mean depth (95% CI) of 190 μm (186 to 195 μm) in L2/3, 667 μm (653 to 681 μm) in L6a, and 843 μm (812 to 872 μm) in L6b. After calcium imaging, a high-resolution z-resolution volume image of the whole cortical depth (z-step = 2 or 3 μm) was acquired. For chronic two-photon Ca^2+^ imaging, the corresponding area of the previous imaging session was identified using the vascular pattern of the brain surface and the location of the neurons. If the vascular pattern changed markedly or if significant bleeding was observed in the cranial window, further imaging was stopped.

Registration and cell detection were performed using Suite2p (52). To correct for neuropil contamination from the signals in somata, the fluorescence time series of neurons ( $F_{soma}$ ) was subtracted from the associated neuropil signal ( $F_{neuropil}$ ) scaled by contamination factor α as follows: $F_{corrected}=F_{soma}-\alpha F_{neuropil}$ . Contamination factor α was calculated using a robust linear regression of cell fluorescence against neuropil fluorescence (53). If α exceeded 1, α was set to 1. The baseline signal was calculated by applying a sliding median filter (window size of 400 s) to $F_{corrected}$ . Then, $F_{\mathrm{raw}}$ of individual imaging frames was obtained by subtracting the baseline signal from $F_{corrected}$ . The response magnitude in each trial is defined as follows: $\Delta F/F=\left(F-F_{base}\right)/F_{base}$ , where F is the average of $F_{\mathrm{raw}}$ of individual frames during the stimulation period of 4 frames, and $F_{\mathrm{base}}$ is that during the 4 frames before each stimulus.

The number of responsive cells in each layer is summarized in *SI Appendix*, Table S1. Visually responsive cells were defined as those with significantly different responses between the blank and visual stimulus periods in either eye (ANOVA, *P* < 0.01) and when the trial average of ΔF/F to the optimal stimulation exceeded the 5th percentile of the distribution in the FOV (Fig. 1 *D*–*F* and *SI Appendix*, Figs. S2 *B*–*D* and S5). Direction-selective and spatial frequency-selective cells were defined as those with significantly different responses (ANOVA, P < 0.01) across the eight directions (Fig. 1 *G* and *H* and *SI Appendix*, Fig. S2 *E* and *F*) and six spatial frequencies (Fig. 1*I* and *SI Appendix*, Fig. S2*G*), respectively. In OD analysis (Fig. 1*J*), neurons visually responsive to either eye were used. In the analysis of binocular matching (Fig. 1 *L*–*O*), only binocular neurons were used. In the chronic imaging studies (Figs. 3 and 4 and *SI Appendix*, Fig. S8), neurons that showed a significant visual response at least once in either the contralateral or ipsilateral eye stimulation within either imaging session were selected.

The DSI is calculated as follows*:* $\left(R_{pref\;dir}-R_{opposite}\right)/\left(R_{pref\;dir}+R_{opposite}\right)$ , where $R_{pref\;dir}$ is the trial average of ΔF/F in the preferred direction, and $R_{opposite}$ is that in the opposite direction. The OSI is obtained as follows: $\left(R_{pref\;ori}-R_{ortho}\right)/\left(R_{pref\;ori}+R_{ortho}\right)$ , where $R_{pref\;ori}$ is the trial average of ΔF/F to the preferred orientation, and $R_{ortho}$ is the orthogonal orientation. The SSI was defined as follows: $\left(R_{pref\;sf}-R_{min}\right)/R_{pref\;sf}$ , where $R_{pref\;sf}$ is the trial average of ΔF/F to the preferred spatial frequency, and $R_{min}$ is the minimal response of ΔF/F to the presented spatial frequencies. The preferred orientations were calculated using the average vector. The VBI is calculated as follows: 1-dOri/45, where dOri reflects the absolute value of the difference between the preferred orientation and vertical orientation, ranging from −1 (horizontal) to 1 (vertical). The spatial frequency that exhibited a peak response was defined as the preferred spatial frequency. For chronic two-photon Ca^2+^ imaging, the assignment of neurons detected in the first imaging session to the second session was performed semiautomatically using a control point matching method. A bootstrap method was used to verify whether the ODI of each neuron changed between the two imaging sessions (Fig. 4 and *SI Appendix*, Fig. S9). Five to seven ΔF/F values were acquired by random sampling with replacement for each of the 48 grating stimuli. Subsequently, ${\Delta F/F}_{ipsi}$ and ${\Delta F/F}_{cont}$ were obtained, and the ODI was calculated. These procedures were repeated 1,000 times. The 5th and 95th percentiles of bootstrap samples were obtained. The 5th percentile greater than 0 and the 95th percentile less than 0 were defined as neurons with OD shift to the ipsilateral (open) eye and to the contralateral (deprived) eye, respectively.

### Tissue Clearing and 3D Immunostaining.

After in vivo two-photon imaging, mice were deeply anesthetized with isoflurane, perfused transcardially with 25 mM PBS (−), and fixed by perfusing 4% paraformaldehyde (PFA) in 0.1 M phosphate buffer (PB). Dissected brains were postfixed with 4% PFA overnight at 4 °C. An ~2-mm-thick block containing the imaged region was trimmed from the brain sample, and the CUBIC-HV method was performed (35). The fixed brains were washed 3 times in PBS for 1 h at 25 °C and were then incubated in 2 mL 0.5 × CUBIC-L (1:1 dilution with water, T3740, TCI) for 6 h. The solution was thereafter replaced with 2 mL 1× CUBIC-L and dilapidated for 2 d at 37 °C with gentle shaking. The sample was then washed three times in PBS for 2 h at 37 °C and incubated in 10 mM HEPES (#17514-15, Nacalai Tesque), 10%(v/v) Triton X-100 (#12967-45, Nacalai Tesque), 200 mM NaCl, and 0.5%(w/v) casein (#030-01505, Wako) in DW (HEPES-TSC) for 1.5 h at 37 °C. Consequently, the samples were subjected to immunostaining with anti-CTGF goat antibody (1:250; sc-14939, Santa Cruz) and anti-Foxp2 rabbit antibody (1:125; ab16046, Abcam) in 250 μL HEPES-TSC for 5 d at 32 °C, with gentle shaking. The samples were then washed 3 times with 0.1 M PB with Triton X-100 (PBT, 10% v/vTritonX100) for 0.5 h at 32 °C with gentle shaking and incubated in HEPES-TSC for 1.5 h at 32 °C. This was followed by staining with Cy3-conjugated anti-rabbit IgG donkey antibody (1:125, 711-167-003, Jackson ImmunoResearch laboratories) and Fluor Alexa 647-conjugated anti-goat IgG donkey antibody (1:125, 705-605-147, Jackson ImmunoResearch laboratories) in 250 μL HEPES-TSC for 5 d at 32 °C with gentle shaking. The samples were then washed 2 times with 0.1 M PBT for 0.5 h and with 0.1 M PB for 1 h, post-fixed with 1% PFA for 12 h, and washed with 0.1 M PB for 2 h at 25 °C. The samples were then incubated in 0.5× CUBIC-R+ (1:1 dilution with water, T3741, TCI) for 1 h, and then replaced with 1× CUBIC-R+ for 1.5 h at 25 °C with gentle shaking. The samples were embedded in an imaging chamber filled with CUBIC-R+ and sealed with a glass cover.

High-z-resolution whole-cortical volume images (z-step = 2 μm) were acquired using a confocal microscope (FV1000, Olympus) equipped with a water immersion objective lens (20×, NA 0.95, XLUMPlanFI 20xW, Olympus). The excitation (Ex) and emission (Em) wavelengths were as follows: CTB 405: Ex 405 nm, Em 410 to 450 nm; GCaMP: Ex 488 nm, Em 500 to 530 nm; CTB 555 and Cy3: Ex 543 nm, Em 560 to 620 nm; Alexa 647: Ex 633 nm, Em >650 nm. The corresponding area of in vivo imaging was identified using the vascular pattern of the brain surface and the location of the neurons in the cleared brain. A subset of GCaMP(+) neurons in vivo (n = 1,061 cells from 8 mice) was assigned to GCaMP(+) neurons in the cleared brain, and the signal intensities of CTGF and Foxp2 in their somata were measured. The background, which was estimated from the median of each FOV, was subtracted from the signal. Foxp2/CTGF signal index was calculated as follows: (Foxp2 signal intensity − CTGF signal intensity)/(Foxp2 signal intensity + CTGF signal intensity). CTGF-single-positive, Foxp2-single-positive, and CTGF- and Foxp2-double-positive neurons were identified manually. A few neurons identified (21 of 1,061 cells) as CTGF-single-positive and Foxp2-single-positive neurons were 1 and −1 of the Foxp2/CTGF signal index, respectively, probably because of an unreasonable background.

## Statistical Analysis.

All quantification and statistical analyses were performed using MATLAB. The permutation test, Mann–Whitney *U* test, Wilcoxon signed-rank test, and Fisher’s exact test were used. For the permutation test, rearrangement was performed 1,000 times if the number of all possible combinations exceeded the value. The Holm–Bonferroni correction was conducted by comparing more than two groups. Correlations were calculated using Pearson’s correlation coefficients.

## Supplementary Material

Appendix 01 (PDF)

## Data Availability

All study data are included in the article and/or *SI Appendix*.
