# Supplementary material for: Experience-dependent functional plasticity and visual response selectivity of surviving subplate neurons in the mouse visual cortex
Source: Proc Natl Acad Sci U S A. 2023 Feb 22;120(9):e2217011120. doi: 10.1073/pnas.2217011120 (PMC9992851; doi:10.1073/pnas.2217011120)
Supplement: Supplementary file 1 — Appendix 01 (PDF) [file pnas.2217011120.sapp.pdf]

PNAS

Supporting Information for
Experience-dependent functional plasticity and visual response selectivity of surviving cortical subplate neurons

Taisuke Yoneda[1,2]*, Kenji Hayashi[1,2], Yumiko Yoshimura[1,2,3]*

*Corresponding authors
Yumiko Yoshimura, **Email:** yumikoy@nips.ac.jp
Taisuke Yoneda, **Email:** yoneda@nips.ac.jp

This PDF file includes:

Figures S1 to S10
Table S1

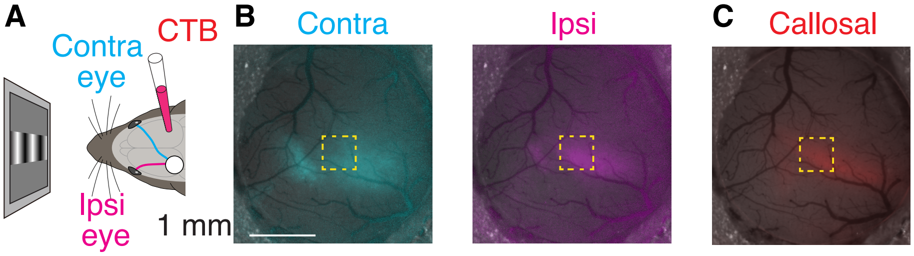

Fig. S1. Identification of binocular area of V1. (A) Scheme of wide-field imaging. A retrograde tracer (cholera toxin B subunit, CTB) is injected into the right V1. Craniotomy is performed on the left V1. Visual stimuli are restricted to the binocular visual field and presented alternately to each eye. (B) Areas responsive to contralateral (Contra, cyan) and ipsilateral (Ipsi, magenta) eye stimulations. Yellow rectangles represent the field of view (FOV) of two-photon imaging. (C) Callosal projection neurons (red) in vascular images.

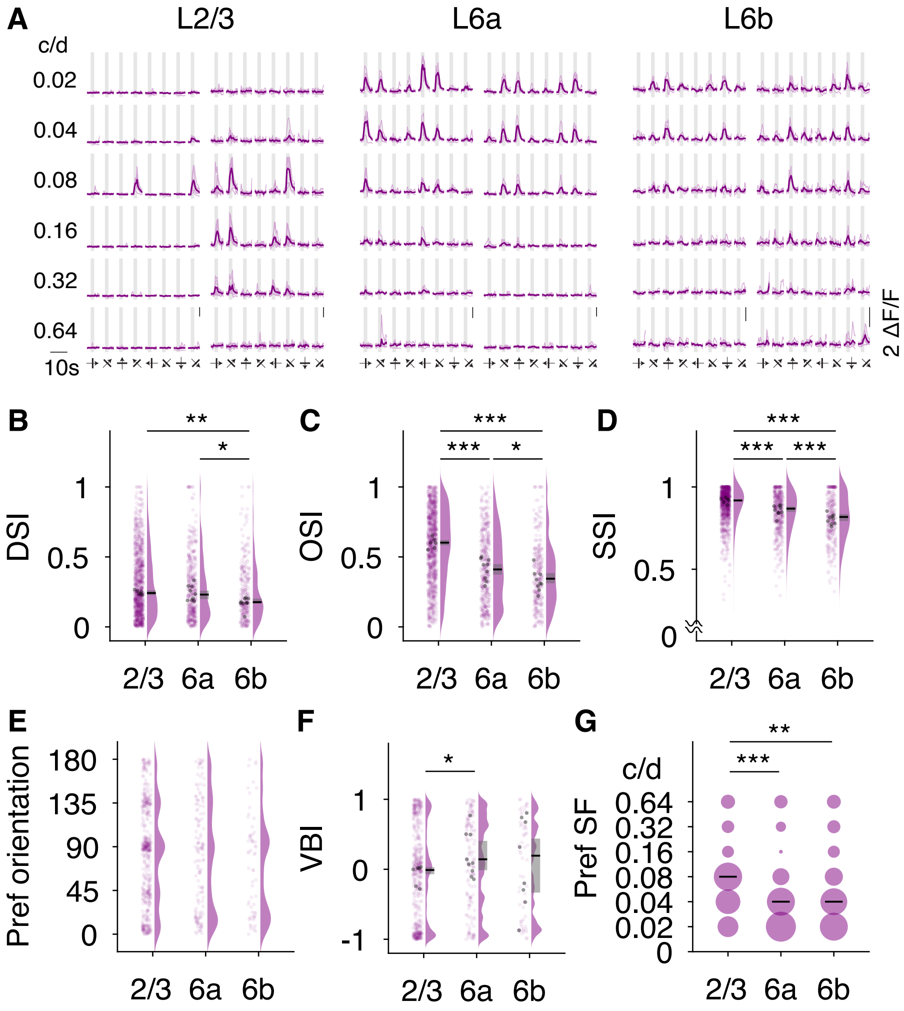

Fig. S2. Receptive field properties of ipsilateral eye responses. (A) Examples of responses to grating stimuli obtained from 2 neurons each in L2/3, L6a, and L6b. Individual (light magenta) and average (magenta) responses are shown. Gray rectangles represent the stimulation period. (B–F) Distribution of direction selectivity index (DSI, B), orientation selectivity index (OSI, C), spatial frequency selectivity index (SSI, D), preferred orientation (E), and vertical bias index (VBI, F) in each layer. Pale-colored and black dots show the values from individual neurons and the mean data of individual mice (B–D, F), respectively. Black lines and gray squares on the kernel density (B–D, F) estimation indicate the mean and 95% confidence interval (CI). (G) Distribution of preferred spatial frequency. The size of the circles shows the density of each spatial frequency. Black lines represent the mean values. The number of animals and the corresponding number of cells are as follows: n = 6 mice (L2/3), 9 mice (L6a), and 9 mice (L6b); (B–D): n = 1003 cells (L2/3), n = 427 (L6a), and n = 287 (L6b); (E and F): n = 456 (L2/3), n = 142 (L6a), and n = 62 (L6b); (G): n = 513 (L2/3), n = 225 (L6a), and n = 110 (L6b). Permutation test (B–D, F) and Mann-Whitney U-test test with Holm-Bonferroni method (G) are performed (*$p < 0.05$, **$p < 0.01$, ***$p < 0.001$). Ipsilateral eye responses in L6b neurons show lower tuning for direction, orientation, and spatial frequency selectivity than those in L2/3 and L6a. Additionally, preferred spatial frequency was lower in L6b than that in L2/3.

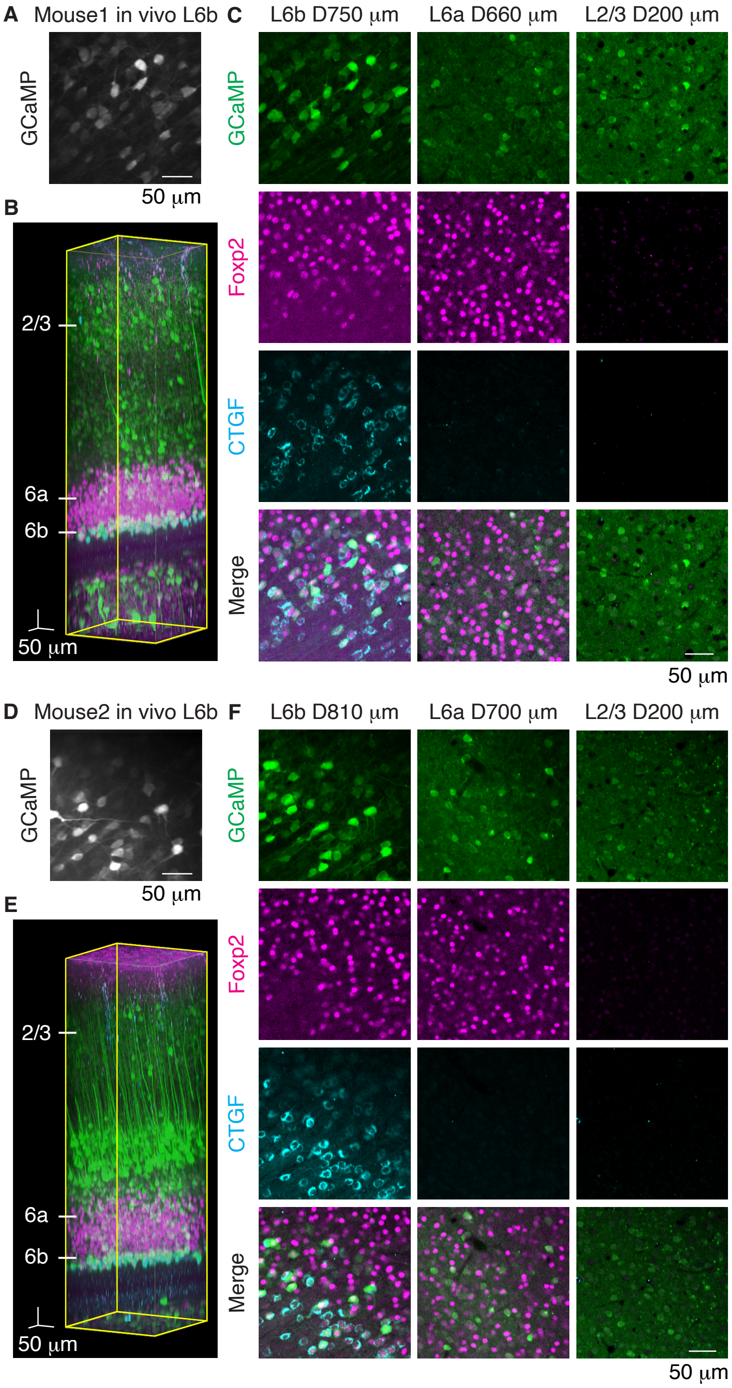

Fig. S3. Identification of subplate neurons after in vivo imaging. (A) An example image of in vivo imaging of L6b. (B) An example of a volumetric image of clearing V1 with CUBIC-HV. Green represents GCaMP signals; magenta and cyan represent immunofluorescence for forkhead box P2 (Foxp2) and connective tissue growth factor (CTGF), respectively. Horizontal white lines on the left indicate the recording depth for each layer. (C) Cross-sections of the volumetric image from layer 6b (L6b), layer 6a (L6a), and layer 2/3 (L2/3). D, Depth from the brain surface after tissue clearing. Foxp2 and CTGF signals were slight in L2/3, Foxp2 signals were prominent in L6a, and Foxp2 and

CTGF signals were strong in L6b. The volumetric image in (B) and the cross-section of L6b in (C) are also shown in Figure 2. (D–F) Example of images of CUBIC-HV from another animal.

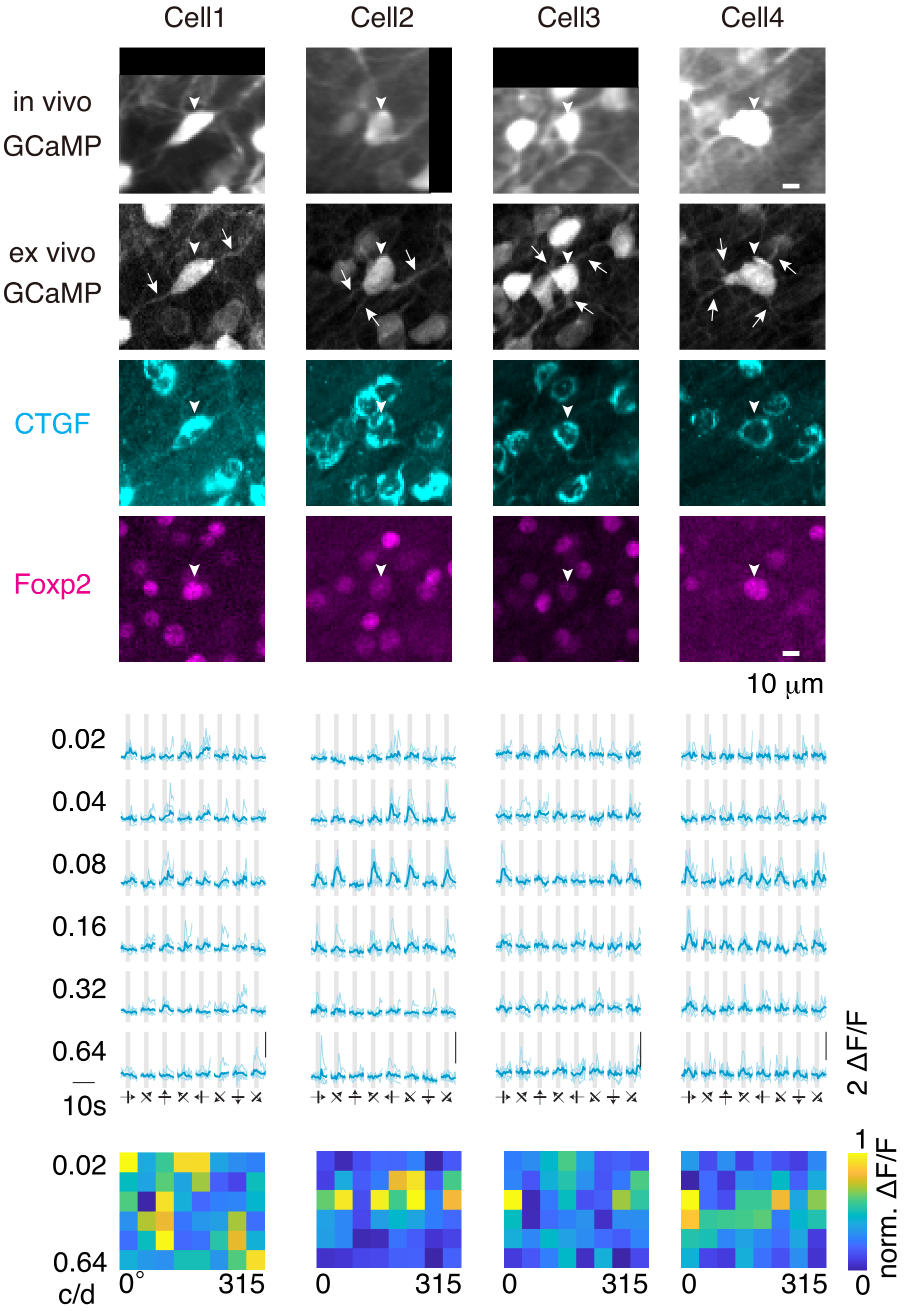

Fig. S4. Morphological features of CTGF-positive L6b neurons. Top: Example images of recorded L6b neurons. Each neuron expressed both CTGF and Foxp2, as indicated by arrowheads. Arrows in ex vivo GCaMP photos show remarkable neurites. Middle: Individual (light blue) and average (dark blue) calcium responses to grating stimuli of 8 directions and 6 spatial frequencies delivered to the contralateral eye. Gray rectangles represent the stimulation period. Bottom: Heatmaps of average responses during the stimulation period. Cell 1 shows bipolar cell-like morphology, and Cell 2–4 exhibit multipolar cell-like morphology.

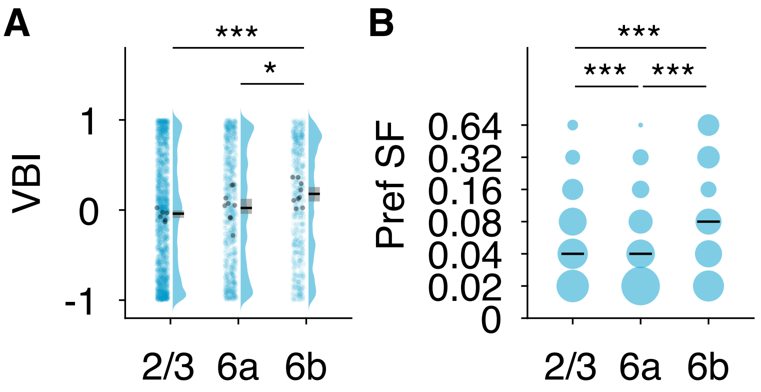

Fig. S5. Preferred orientation and spatial frequency of visually responsive L6b neurons. (A) vertical bias index (VBI) of contralateral eye responses in each layer. Pale-colored and black dots show the values from individual neurons and the mean data of individual mice, respectively. Black lines and gray squares on the kernel density estimation indicate the median and 95% confidence interval (CI). (B) The distribution of preferred spatial frequency. The size of the circles shows the density of each spatial frequency. Black lines represent the median. The number of animals and cells are as follows: n = 6 mice (L2/3), 9 mice (L6a), and 9 mice (L6b); n = 1868 cells (L2/3), n = 847 (L6a), and n = 590 (L6b). Permutation test (A) and Mann-Whitney U-test test with Holm-Bonferroni method (B) are performed (*$p < 0.05$, ***$p < 0.001$).

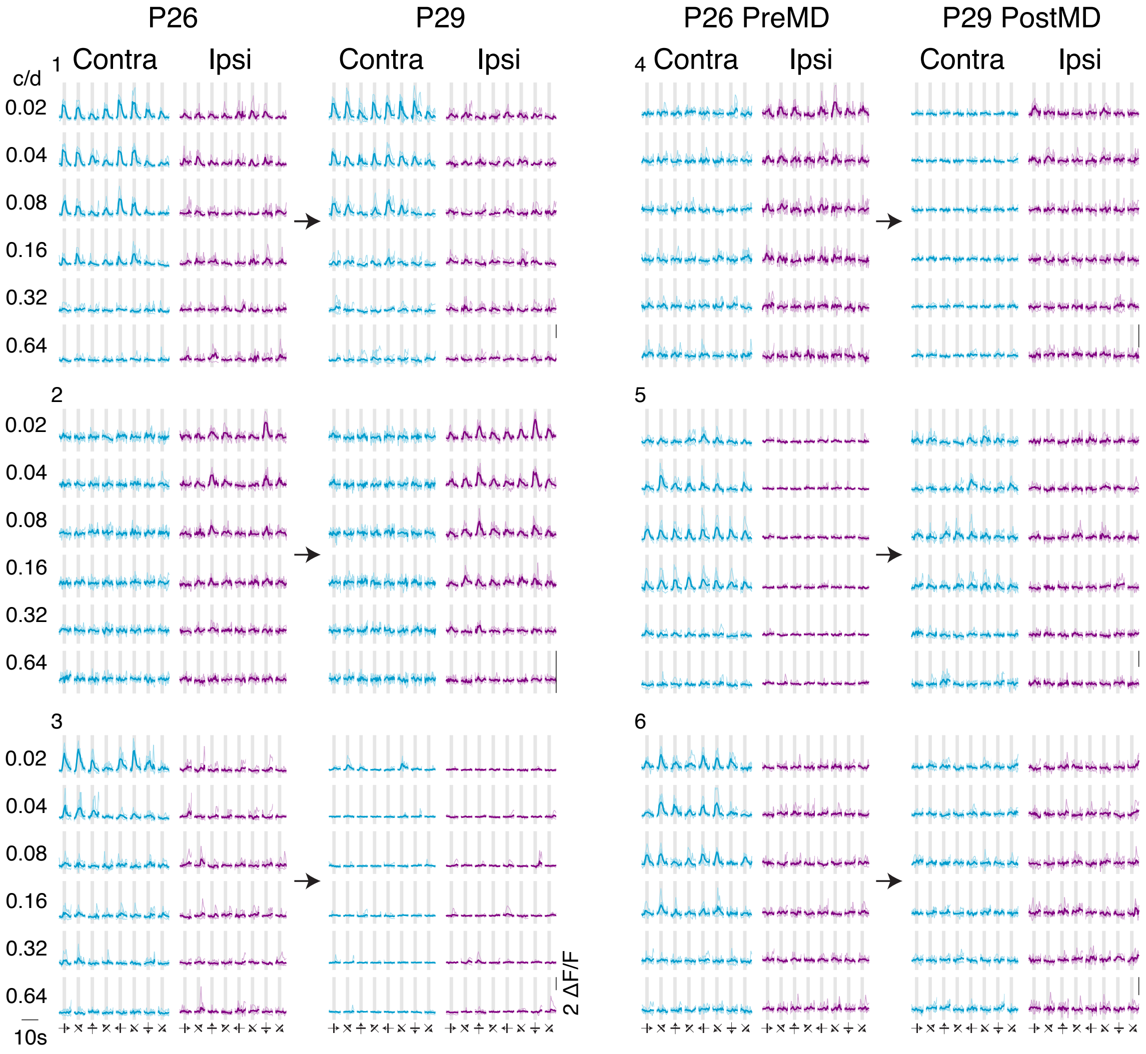

Fig. S6. Examples of Ca^{2+} responses of the chronically recorded L6b neurons shown in Figure 3A. Numbers in the upper left correspond to those in Figure 3A. Individual (pale color) and average (dark color) responses to grating stimuli delivered to the contralateral (Contra) and ipsilateral eye (Ipsi) in each imaging session. Gray rectangles on the top panels represent the stimulation period.

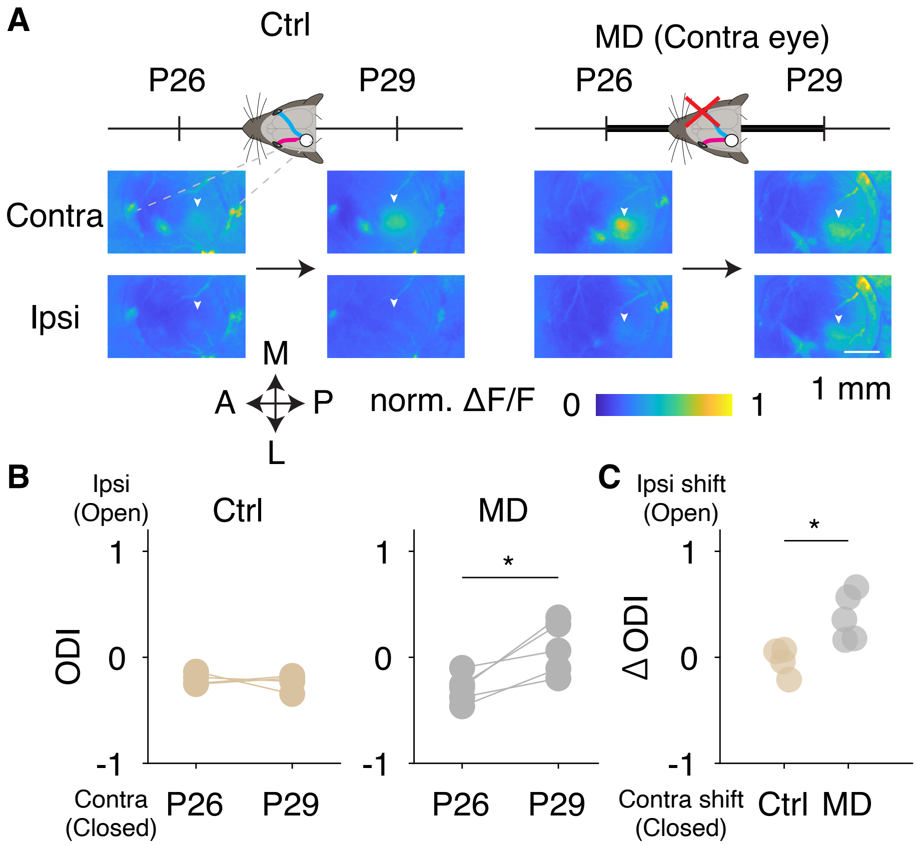

Fig. S7. OD plasticity evaluated by chronic wide-field Ca^{2+} imaging. (A) Examples of the imaging at P26 and P29 in control (Ctrl, left) and monocular-deprived (MD) mice (right). Color shows normalized fluorescent changes during visual stimuli to the contralateral (Contra) or ipsilateral (Ipsi) eye. White arrowheads indicate the binocular area of V1. In MD mice, ipsilateral eye was non-deprived eye, and contralateral eye was deprived eye. A, anterior; P, posterior; L, lateral; M, medial. (B) ODI before and after MD (n = 4 for Ctrl, n = 5 for MD) assessed by the fluorescent changes in the binocular region. Each circle represents the data of an individual mouse, and pairs connected by lines represent the same animal. (C) Distribution of the difference in ODI (ΔODI) between the two imaging sessions. Each circle represents the data of an individual mouse. Wilcoxon signed-rank test is performed (*$p < 0.05$). MD mice show a significant shift of OD to the open eye.

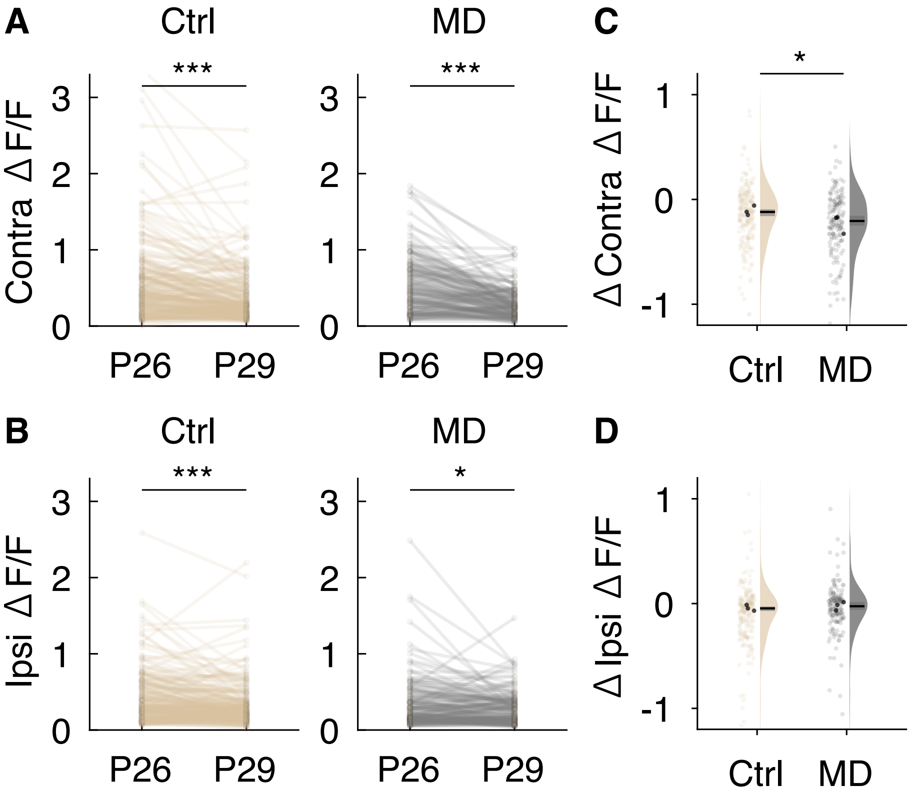

Fig. S8. Response amplitude in L6b neurons with chronic two-photon imaging. (A and B) The distribution of response amplitude to the contralateral eye stimulation (Contra eye ΔF/F, A) and that to the ipsilateral eye stimulation (Ipsi eye ΔF/F, B) in the control (left, n = 216 cells from 3 mice) and monocular-deprived (MD) mice (right, n = 156 cells from 3 mice). In MD mice, ipsilateral eye was non-deprived (open) eye, and contralateral eye was deprived (close) eye. The same neurons between two imaging sessions are connected with lines. (C and D) The difference in the response amplitude of the contralateral and ipsilateral eyes between the two imaging sessions. Positive and negative values indicate potentiation and depression of response magnitude at P29, respectively. Pale and black circles show the values of individual neurons and the mean data of individual mice, respectively. Black lines and gray squares on the kernel density estimation indicate the median and 95% CI. Mann-Whitney U-test test (A and B) and permutation test (C and D) are performed (*p < 0.05, ***p < 0.001). MD induced a significant depression in the response to the deprived eye.

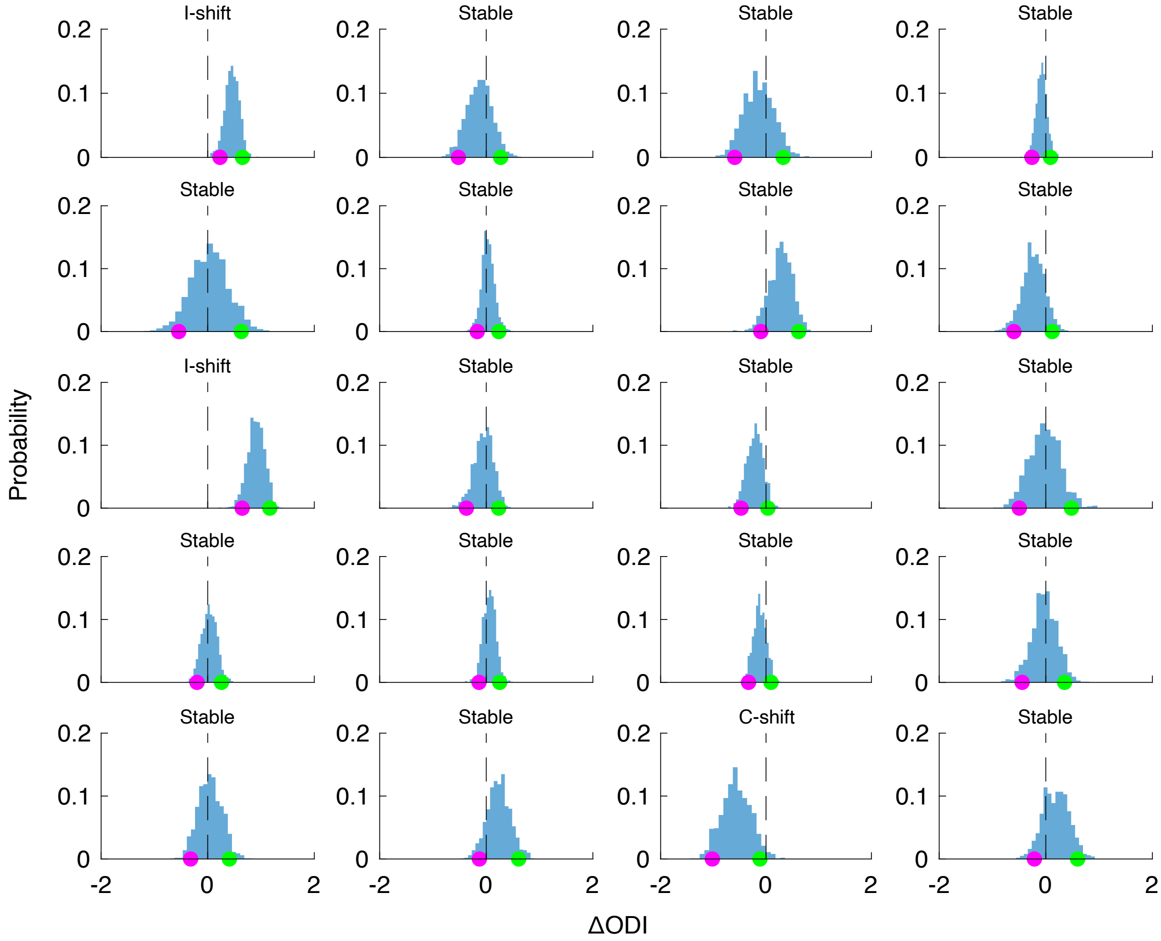

Fig. S9. Histograms represent bootstrap samples of the distribution of the ΔODI in each L6b neuron. Magenta and green circles represent the 5th and 95th percentiles of the distribution, respectively. I-shift, the ODI is shifted toward ipsilateral eye. C-shift, the ODI is shifted toward contralateral eye.

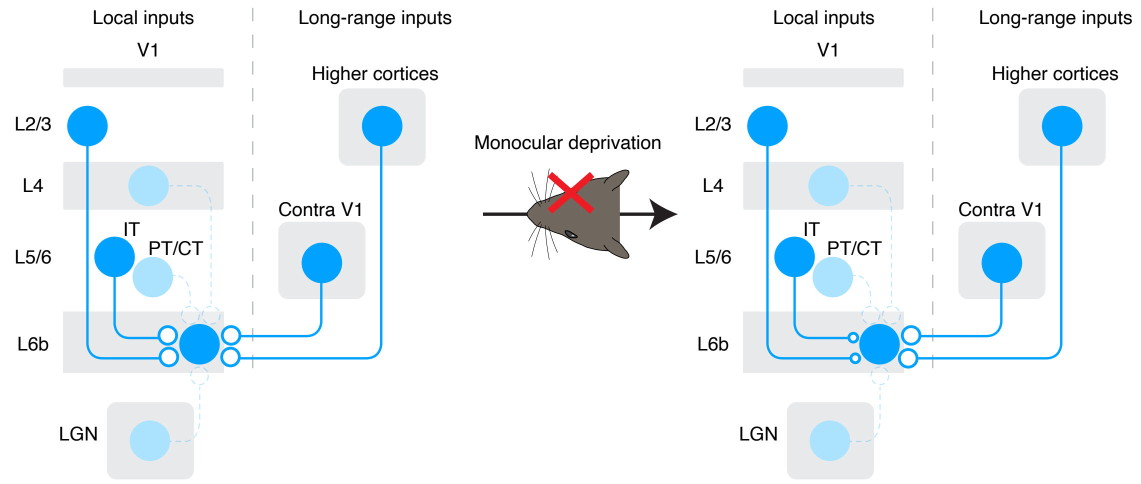

Fig. S10. Potential circuit mechanisms underlying ocular dominance plasticity of L6b neurons. Blue and white circles indicate neurons and synapses, respectively. White circle size reflects synaptic strength. IT, intratelencephalic; PT, pyramidal tract; CT, corticothalamic; LGN, lateral geniculate nucleus.

Table S1. The number of responsive cells.

Reponsive cell group	Eye	Cohort	n(animals)	Group/Total	Proportion	Figure
Visually responsive	Contralateral	L2/3	6	1868 / 2522	74.1%	Fig. 1D-F, Fig. S5
		L6a	9	847 / 1807	46.9%	Fig. 1D-F, Fig. S5
		L6b	9	590 / 1386	42.6%	Fig. 1D-F, Fig. S5
Direction selective	Contralateral	L2/3	6	1086 / 2522	43.1%	Fig. 1G and H
		L6a	9	356 / 1807	19.7%	Fig. 1G and H
		L6b	9	156 / 1386	11.3%	Fig. 1G and H
Spatial frequency selective	Contralateral	L2/3	6	1261 / 2522	50.0%	Fig. 1I
		L6a	9	510 / 1807	28.2%	Fig. 1I
		L6b	9	313 / 1386	22.6%	Fig. 1I
Visually responsive	Either eye	L2/3	6	2052 / 2522	81.4%	Fig. 1J
		L6a	9	1031 / 1807	57.1%	Fig. 1J
		L6b	9	686 / 1386	49.5%	Fig. 1J
Visually responsive	Binocular	L2/3	6	819 / 2522	32.5%	Fig. 1L-O
		L6a	9	243 / 1807	13.4%	Fig. 1L-O
		L6b	9	191 / 1386	13.8%	Fig. 1L-O
Visually responsive	Contralateral	L6b CTGF+	8	175 / 380	46.1%	Fig. 2G-K, M-Q
		L6b CTGF+Foxp2+	8	175 / 435	40.2%	Fig. 2G-K, M-Q
		L6b Foxp2+	8	56 / 220	25.5%	Fig. 2G-K, M-Q
Visually responsive on P26 or P29	Either eye	L6b Control	3	216 / 325	66.5%	Fig. 3,4, and Fig. S8
		L6b MD	3	156 / 282	55.3%	Fig. 3,4, and Fig. S8
Visually responsive	Ipsilateral	L2/3	6	1003 / 2522	39.8%	Fig. S2B-D
		L6a	9	427 / 1807	23.6%	Fig. S2B-D
		L6b	9	287 / 1386	20.7%	Fig. S2B-D
Direction selective	Ipsilateral	L2/3	6	456 / 2522	18.1%	Fig. S2E and F
		L6a	9	142 / 1807	7.9%	Fig. S2E and F
		L6b	9	62 / 1386	4.5%	Fig. S2E and F
Spatial frequency selective	Ipsilateral	L2/3	6	513 / 2522	20.3%	Fig. S2G
		L6a	9	225 / 1807	12.5%	Fig. S2G
		L6b	9	110 / 1386	7.9%	Fig. S2G
